# Co-aminobenzamid@Al-SBA-15: a favorable catalyst in synthesis of 2,3-dihydroquinazolin-4(1*H*)-ones

**DOI:** 10.1186/s13065-019-0517-7

**Published:** 2019-02-28

**Authors:** Javad Safaei-Ghomi, Raheleh Teymuri, Atefeh Bakhtiari

**Affiliations:** Department of Organic Chemistry, Faculty of Chemistry, University of Kashan, Kashan, P.O. Box 87317-51167, Iran

**Keywords:** Modified mesoporous, Aluminosilicate, Dihydroquinazolinones, Three-component condensation

## Abstract

The novel hybrid Co-aminobenzamid@Al-SBA-15 was synthesized as a modified mesoporous catalyst. To achieve this aim, the obtained materials were investigated by various techniques including Fourier transform infrared spectroscopy (FT-IR), X-ray diffraction (XRD), N_2_ adsorption–desorption, field emission scanning electron microscopy (FE-SEM) and transmission electron microscopy (TEM) to examine the functional groups, crystallinity, surface area, morphology, particle size distribution and amounts of functional groups, respectively. Co-aminobenzamid@Al-SBA-15 exhibits excellent catalytic activity for the synthesis of 2,3-dihydroquinazolin-4(1*H*)-ones under mild reaction conditions. The heterogeneous catalyst showed good recyclability and can be reused for ten consecutive cycles without significant loss of its catalytic activity.

**Electronic supplementary material:**

The online version of this article (10.1186/s13065-019-0517-7) contains supplementary material, which is available to authorized users.

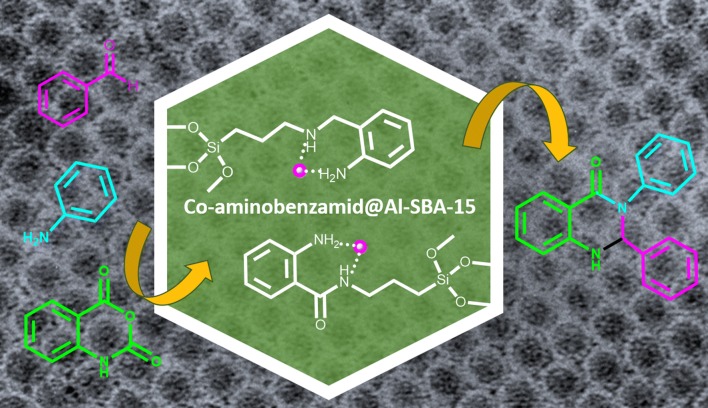

## Introduction

Nitrogen-containing fused-heterocycles are an integral part of biological and small molecule drugs or synthetic molecules [1] and physiologically active natural products [2]. Noteworthy, one of this fused-heterocycle is 2,3-dihydroquinazolinone (DHQZ-1) which contains wide pharmacological properties including anti-inflammatory, antibacterial, antitumor, and anticonvulsant [3–6]. Having looked at this importance, various catalysts were employed including, KAl(SO_4_)_2_·12H_2_O [7], silica sulfuric acid (SSA) [8], aluminum methanesulfonate [9], ZnO nanoparticles [10], Al(H_2_PO_4_)_3_ [11], Montmorillonite K-10 [12], β-Cyclodextrin [13], Co(m-NBS)_2_ [14]. Whereas the majority of these procedures have noticeable negative aspects such as long reaction times, low yields, harsh reaction conditions, and use of expensive and toxic catalysts. Therefore, to avoid these limitations, the exploration of an efficient, easily available catalyst with high catalytic activity and short reaction times for the preparation of dihydroquinazolins is still favored.

**Scheme 1:**
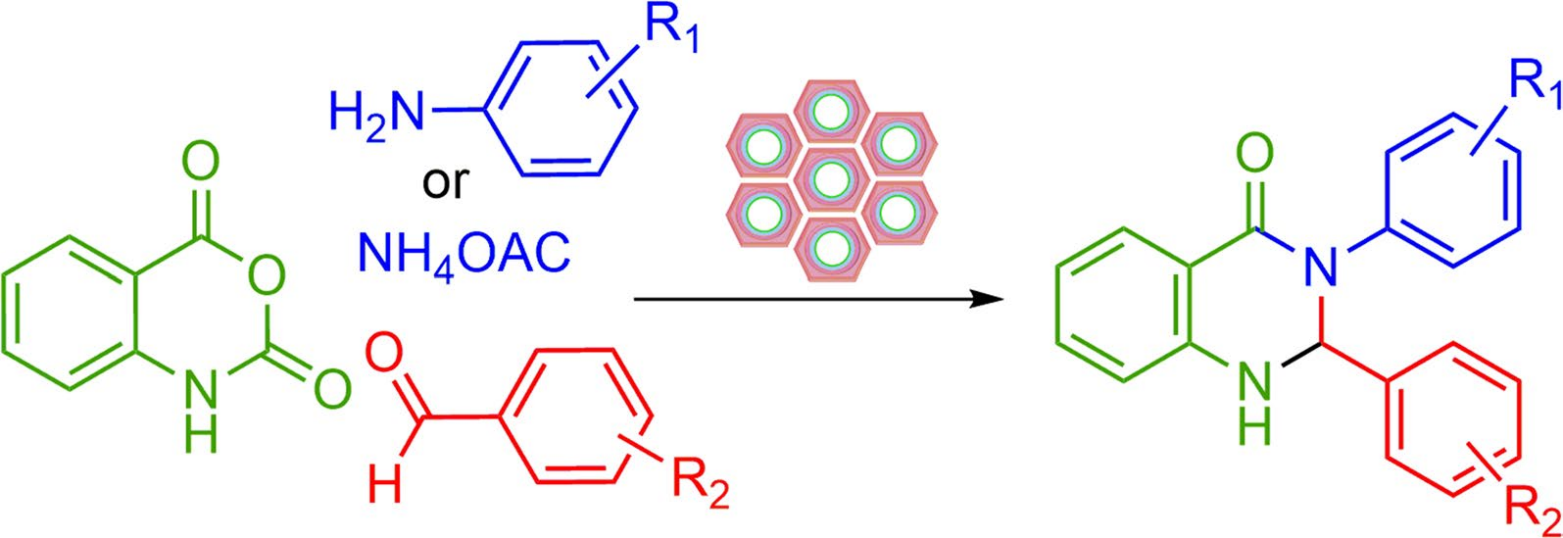
Co-aminobenzamid@Al-SBA-15 catalyzed the synthesis of 2,3-dihydroquinazolin-4(1*H*)-one derivatives

Ordered mesoporous silicas such as those of M41S, SBA-n, and MSU-X families discovered in the early 1990s have been regarded as an encouraging class of materials for separation and catalysis [15, 16]. These materials serve as an unprecedented choice for such applications. In fact, they offer high specific surface areas, large and defined pore sizes, defined surface acidity, and excellent mechanical and thermal stability [17, 18]. Moreover, the substituents such as aluminum, titanium, and zirconium can be incorporated into the silica framework to obtain materials for applications such as catalysis and ion exchange. Among the metal substituted mesoporous materials, aluminum-incorporated mesoporous materials have considerable potential in moderating acid-catalyzed reactions for large molecules [19–21]. A large number of research groups have sought new and novel approaches toward incorporating multiple functional groups onto heterogeneous catalysts, which can catalyze multistep reaction cascades in one system or work in a cooperative manner to alter the characteristics of a single reaction [22–24]. In the current work, the use of material with surface functional groups shows improved selectivity catalyst. Consequently, we synthesize the novel hybrid Co-aminobenzamid@Al-SBA-15 by amino-functionalized with employing 3-aminopropyltriethoxysilane (3-APTES) and anchored 2-aminobenzamide on it. The synthesizing process pursued by grafting of Co (II) to catch the desired product. Also, the activity of catalyst has been scrutinized by synthesizing 2,3-dihydroquinazolin-4(1*H*)-one derivatives (Scheme 1).

## Results and discussion

**Fig. 1:**
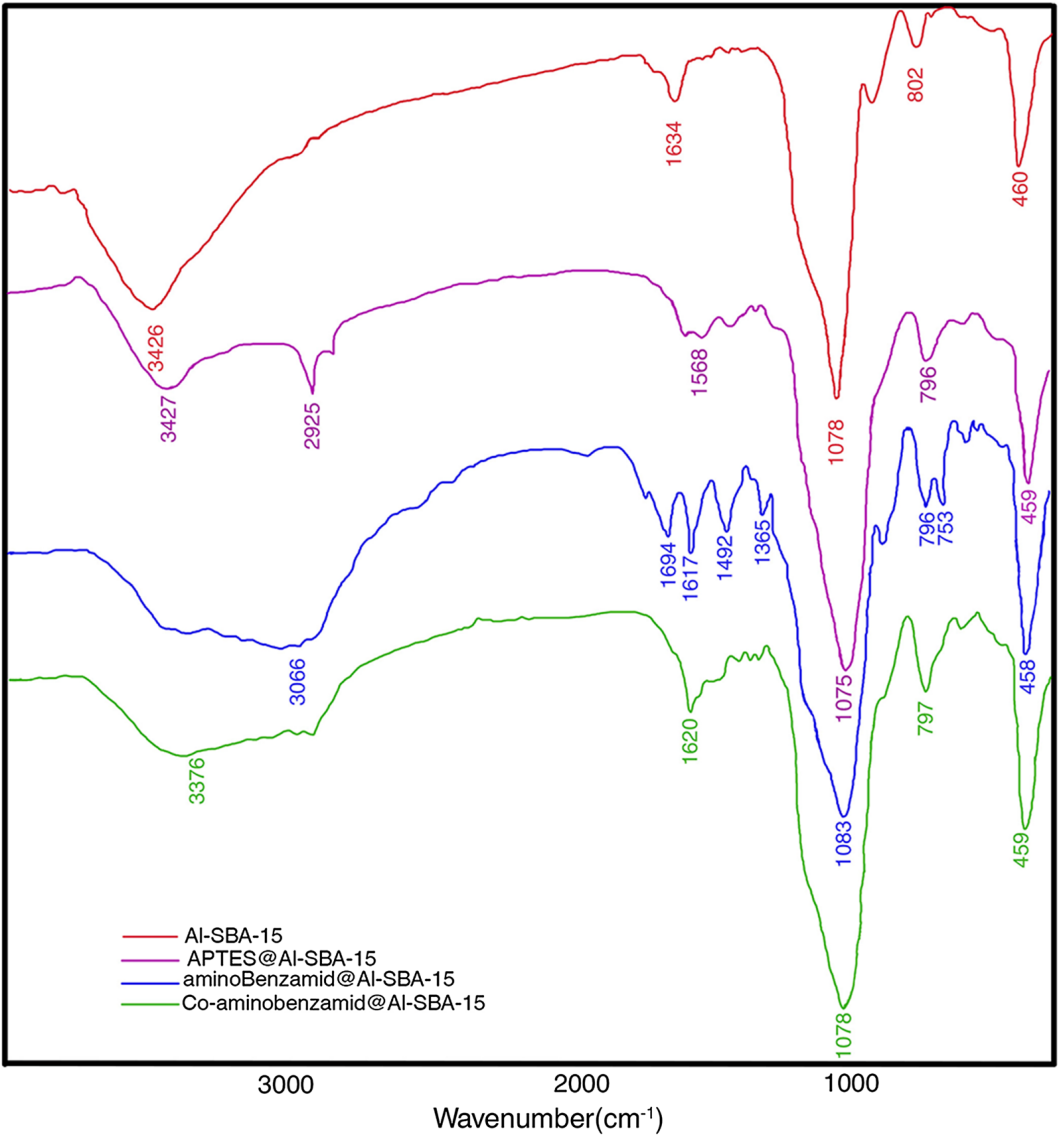
Fourier-transform infrared (FT-IR) spectra of Al-SBA-15, ATPES@Al-SBA-15, aminobenzamid@Al-SBA-15, and Co-aminobenzamid@Al-SBA-15

The Fourier-transform infrared (FT-IR) spectra of Al-SBA-15, APTMS@Al-SBA-15, aminobenzamid@Al-SBA-15, and Co-aminobenzamid@Al-SBA-15 are shown in Fig. 1. The peaks at 600–1200 cm^−1^ can be attributed to the vibration of Si–O groups in the mesoporous silica framework. The absorption bands of Al-SBA-15 based materials at 1078, 802, and 460 cm^−1^ are attributed to the Si–O–Si anti-stretching vibration, the Si–O–Si stretching vibration, and the bending vibration of Si–O, respectively. In the FT-IR spectrum of APTES@Al-SBA-15, there are the characteristic bands of –NH_2_ at 3427, 1568 cm^−1^. For the aminobenzamid@Al-SBA-15 sample, the absorption peak at 1694, 1617 and 1494 cm^−1^ is observed and attributed to the characteristic peaks of aminobenzamid, due to the presence of the C=O bonds. After aminobenzamid@Al-SBA-15 coordinated with Co, this IR absorption peak shifted from 1617 to 1620 cm^−1^, which is indicative for the formation of a Co–ligand bond. The results above imply the presence of aminobenzamid bonded on the surface of Al-SBA-15, and that the molecular structure of these functional moieties can be perfectly retained in the complex of Co-aminobenzamid@Al-SBA-15.

**Fig. 2:**
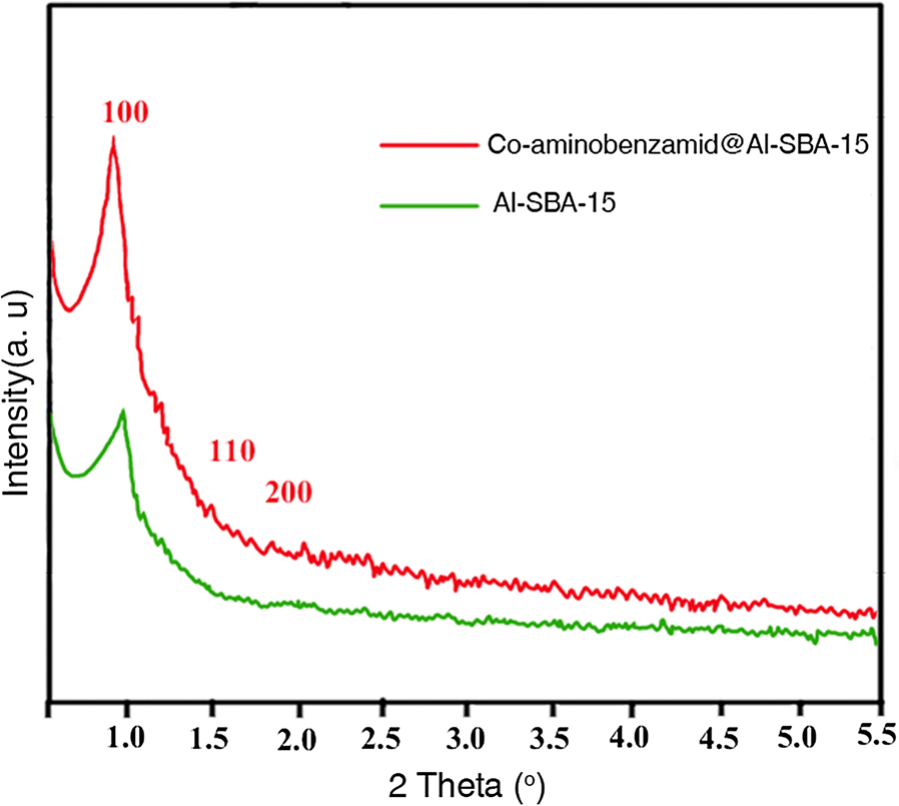
N_2_ sorption isotherms of Al-SBA-15, aminobenzamid@Al-SBA-15, and Co-aminobenzamid@Al-SBA-15

The N_2_ adsorption/desorption isotherms and pore-size distribution curves of the samples are displayed in Fig. 2. As shown in Fig. 2, all the isotherms exhibited a typical type IV isotherm with an H1 hysteresis loop starting from P/P_0_ = 0.6. This is the characteristic of mesoporous Al-SBA-15 with ordered pore structures, which is quite important to disperse and stabilize the supported Cobalt species. Compared with the BET surface area (815 m^2^/g) of Al-SBA-15, the surface area of Co-aminobenzamid@Al-SBA-15 was decreased to 581 m^2^/g after Al-SBA-15 was functionalized. The inset displays the narrow pore size distribution centered on 6.42, 4.27 and 3.94 nm for Al SBA-15, aminobenzamid@Al-SBA-15 and Co-aminobenzamid@Al-SBA-15 samples, respectively. Aminobenzamid@Al-SBA-15 displays a little smaller pore size than the parent Al-SBA-15 due to the incorporation of Ph-groups and NH_2_ into the pore channels. These results are in excellent agreement with the fact that the surface of mesoporous Al-SBA-15 has been successfully modified by aminobenzamid. Co species have entered into the channels of the Al-SBA-15 materials, resulting in the decrease in its pore size.

**Fig. 3:**
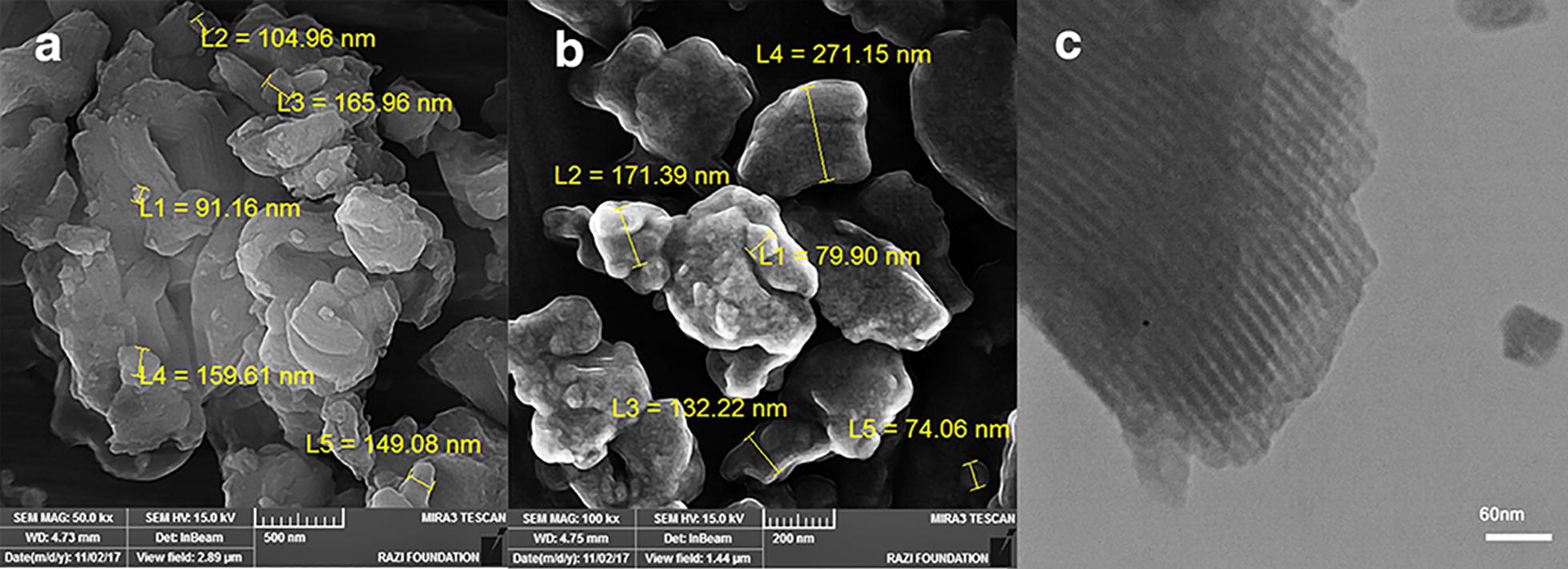
FE-SEM images of **a** Al-SBA-15, **b** Co-aminobenzamid@Al-SBA-15 and TEM image of **c** Co-aminobenzamid@Al-SBA-15

In order to obtain the morphology and particle size of nanoparticles, SEM images of the mesoporous were obtained and are presented in (Fig. 3). As shown in the SEM images, of Fig. 3a the Al-SBA-15 sample is that with the bagel-shaped particles with relatively uniform sizes. After being functionalized with aminobenzamid and Co the shape of Al-SBA-15 is unchanged noticeably (Fig. 3b). The TEM image of the Co-aminobenzamid@Al-SBA-15 sample (Fig. 3c) reveals that no cobalt can be observed in the pores, which shows the Co^2+^ ions have coordinated with two N atoms in aminobenzamid@Al-SBA-15.

**Fig. 4:**
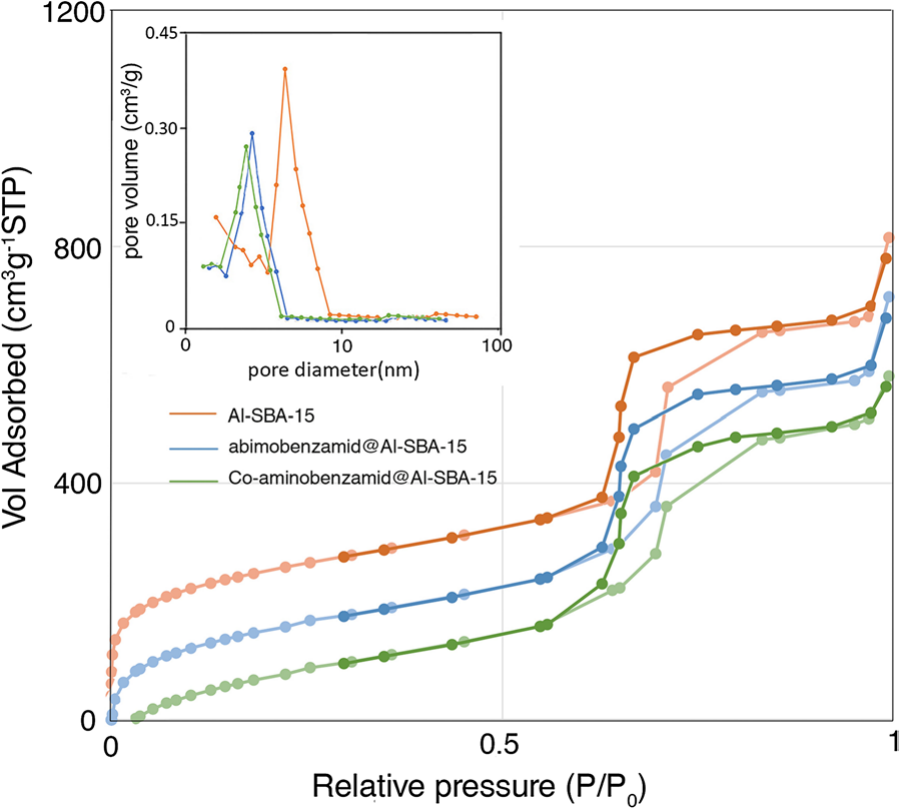
Low angle XRD patterns of Al-SBA-15 and Co-aminobenzamid@Al-SBA-15

**Fig. 5:**
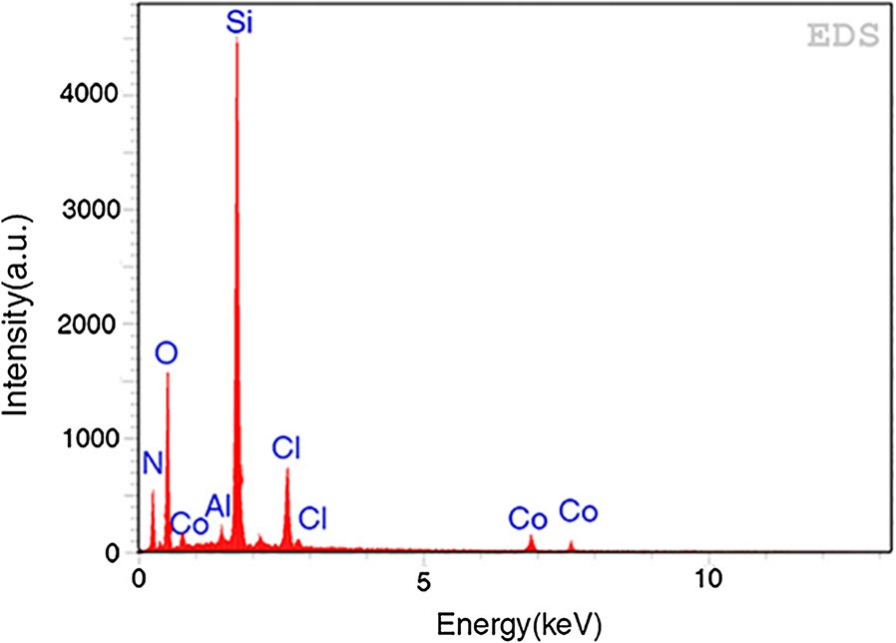
Energy-dispersive X-ray spectroscopy of Co-aminobenzamid@Al-SBA-15

The small-angle XRD patterns of Al-SBA-15 and Co-aminobenzamid@Al-SBA-15 are shown in Fig. 4. The Bragg peaks in the 2q range of 0.8–2, which can be indexed as (1 0 0), (1 1 0) and (2 0 0) reflections of the two-dimensional hexagonal structure of the SBA-15 material. As can be seen, the regularity of Al-SBA-15 is decreased. The ordered structure of Co-aminobenzamid@Al-SBA-15 has remained intact, as supported by the XRD results. Also, there is no major change in the crystallinity of Al-SBA-15 after functionalization and Co immobilization. Furthermore, the diffraction peaks of Co species cannot be detected, which also shows that the Co species were immobilized into the pore channels of Al-SBA-15 in the atom dispersion, and no crystal Co species existed in the sample. The elemental composition of Co-aminobenzamid@Al-SBA-15 spinel nanocrystals was shown by the EDS spectrum (Fig. 5).

Elemental analysis results showed that the carbon, nitrogen, oxygen, aluminum, silicon, cobalt amount of the Co-aminobenzamid@Al-SBA-15 was 25.89, 7.27, 40.92, 1.28, 20.21, 4.43 (wt%).

As a result of our great interest in preparation of heterocyclic compounds by applying heterogeneous catalysts [25, 26], herein we wish to report an efficient procedure to synthesize 2,3-dihydroquinazolin-4(1*H*)-one derivatives through a three-component one-pot condensation of isatoic anhydride, aromatic aldehyde and primary amines/or ammonium acetate using Co-aminobenzamid@Al-SBA-15 as catalyst (Scheme 1).

Beforehand, the reaction of isatoic anhydride, benzaldehyde and aniline were picked out as a model reaction. And now, the effect of experimental factors comprising the type and amount of catalyst and solvent were investigated to find the best condition for this reaction and the results are listed in Table 1. To begin with the solvent examination, it was demonstrated that EtOH is the most effective condition for this condensation of isatoic anhydride, aromatic aldehyde and primary amines or ammonium acetate (Table 1, entry 8). Also, amount of catalysts were investigated in the reaction of isatoic anhydride, benzaldehyde and aniline as listed in Table 1. The highest yield was obtained by 0.03 g of Co-aminobenzamid@Al-SBA-15 under reflux in EtOH.

**Table 1:** The effect of reaction condition on the synthesis of 2,3-diphenyl-2,3-dihydroquinazolin-4(1*H*)-one under various conditions
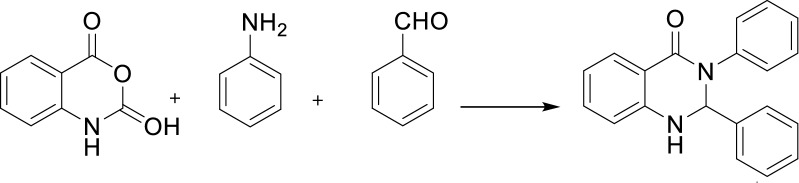

Entry	Solvent (condition)	Catalyst	Time (min)	Yield^a^ (%)
1	Solvent-free	Co-aminobenzamid@Al-SBA-15 (0.03 g)	80	40
2	Water (reflux)	Co-aminobenzamid@Al-SBA-15 (0.03 g)	80	55
3	DCM (reflux)	Co-aminobenzamid@Al-SBA-15 (0.03 g)	60	Trace
4	Acetone (reflux)	Co-aminobenzamid@Al-SBA-15 (0.03 g)	60	25
5	Acetonitrile (reflux)	Co-aminobenzamid@Al-SBA-15 (0.03 g)	90	30
6	Methanol (reflux)	Co-aminobenzamid@Al-SBA-15 (0.03 g)	50	70
7	Ethanol (reflux)	Co-aminobenzamid@Al-SBA-15 (0.02 g)	25	85
8	Ethanol (reflux)	Co-aminobenzamid@Al-SBA-15 (0.03 g)	25	94
9	Ethanol (reflux)	Co-aminobenzamid@Al-SBA-15 (0.05 g)	25	93

^a^ Isolated yields

After optimization of the reaction conditions, to investigate the efficiency and the scope of the presented procedure, numerous mono and disubstituted 2,3-dihydroquinazolin-4(1*H*)-ones were formed via one-pot three-component condensation reactions between isatoic anhydride, aromatic aldehyde and primary amines/or ammonium acetate using catalytic amounts of Co-aminobenzamid@Al-SBA-15 under reflux conditions in ethanol (Tables 2, 3).

**Table 2:** Synthesis of monosubstituted 2,3-dihydroquinazoline-4(1*H*)-ones using Co-aminobenzamid@Al-SBA-15

Entry	Product	Time (min)	Yield^a^ (%)	m.p °C [refs]
**1**		20	96	218–220 [7]
**2**		22	90	214–215 [11]
**3**		20	92	199–201 [7]
**4**		16	97	233–235 [7]
**5**		26	89	192–193 [27]
**6**		25	92	203–205 [9]
**7**		18	95	182–184 [28]
**8**		22	94	204–206 [11]
**9**		15	95	177–179 [7]
**10**		15	92	209–211 [7]

^a^ Isolated yields

**Table 3:** Synthesis of disubstituted 2,3-dihydroquinazoline-4(1*H*)-ones using Co-aminobenzamid@Al-SBA-15

Entry	Product	Time (min)	Yield^a^ (%)	m.p °C [refs]
1		25	94	205–207 [29]
2		28	90	193–195 [7]
3		30	90	217–219 [29]
4		24	96	212–214 [30]
5		28	88	184–185 [9]
6		28	92	216–218 [11]
7		25	95	211–213 [28]
8		38	87	191–192
9		25	92	156–157
10		23	94	209–211 [9]

^a^ Isolated yields

To compare the efficiency of Co-aminobenzamid@Al-SBA-15 with the reported catalysts for the synthesis of 2,3-dihydroquinazoline-4(1*H*)-ones derivatives, we have tabulated the results in Table 4. As Table 4 indicates, Co-aminobenzamid@Al-SBA-15 is superior with respect to the reported catalysts in terms of reaction time, yield and conditions. In addition, our catalyst was recyclable for at least ten times. High catalytic activity and ease of recovery from the reaction mixture, and several reuse times without significant losses in performance are additional eco-friendly attributes of this catalytic system.

**Table 4:** Comparison of catalytic activity of Co-aminobenzamid@Al-SBA-15 with other reported catalysts

Entry	Solvent (condition)	Catalyst	Time (min)	Yield^a^ (%)	[Refs]
1	Ethanol (reflux)	Co-aminobenzamid@Al-SBA-15 (0.03 g)	25	94	This work
2	Ethanol (reflux)	Al-SBA-15 (0.03 g)	45	80	This work
3	EtOH (reflux)	Montmorillonite K-10 (0.3 g)	390	80	[12]
4	H_2_O (reflux)	β-Cyclodextrin (0.2%)	180	84	[13]
5	Ethanol/H_2_O (reflux)	Co(m-NBS)_2_ (3%)	120	97	[14]
6	Ethanol (reflux)	KAl(SO_4_)_2_·12H_2_O (0.5%)	240	88	[7]
7	Solvent-free	Silica sulfuric acid (20%)	300	80	[8]
8	Solvent-free	Al(H_2_PO_4_)_3_ (16%)	35	80	[11]
9	Solvent-free	Nano ZnO (20%)	180	88	[10]

^a^ Isolated yields

As a result of this table, condensation of aniline with commercially available aromatic aldehydes having electron-donating and electron withdrawing substituents produced 4k–t in high yields (Table 3, entries 1–10). According to the results, aldehydes bearing electron-donating groups produced the desired products more quickly than the aldehydes with electron-withdrawing groups. Though meta- and para substituted aromatic aldehydes reacted quickly, ortho substituted aromatic aldehydes give the product in longer reaction time (Table 2, entry 5, 6). Also, the results shown in Tables confirm that the reaction was compatible successfully with a broad range of substituents (both electron-donating and electron-withdrawing groups) in the amines or the aldehydes. All novel and known products were characterized by comparing their physical data, ^1^H, ^13^CNMR, FT-IR spectroscopy, and elemental analysis spectra.

**Scheme 2:**
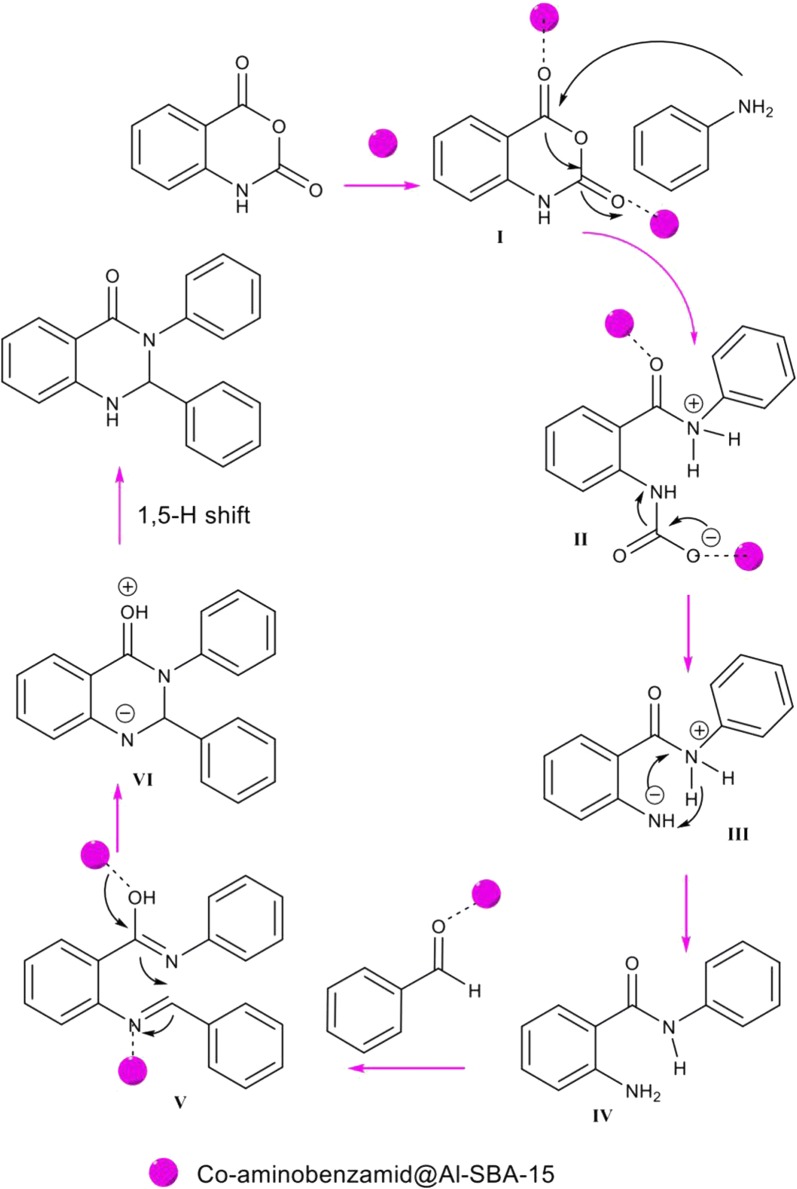
The proposed reaction mechanism for the formation of substituted 2,3-dihydroquinazoline-4(1*H*)-ones using Co-aminobenzamid@Al-SBA-15

On the basis of the point mentioned above, a reasonable mechanism for the preparation of 2,3-dihydroquinazolin-4(1*H*)-ones derivatives by the Co-aminobenzamid@Al-SBA-15 is suggested in Scheme 2. The first point, interaction of Co-aminobenzamid@Al-SBA-15 as a catalyst and isatoic anhydride to produce a reactive intermediate I. And now, the N-nucleophilic primary amine attacks on the carbonyl unit of I to produce a reactive intermediate II, which in turn affords III through decarboxylation reaction. Subsequently, the proton transfer of III affords 2-amino-*N*-substituted-amide IV. Besides the reaction of an activated aldehyde with IV proceeds to produce the imine intermediate V. The part of amide functional group in intermediate IV could be formed using tautomerism phenomenon in the presence of the Co-aminobenzamid@Al-SBA-15. Accordingly, intermediate VI could be prepared by an intermolecular nucleophilic attack of the amide nitrogen on activated imine carbon, followed by a 1,5-proton transfer to yield the final 2,3-dihydroquin-azoline-4-(1*H*)-ones as the concluding product. These steps are efficiently carried out on the high nanoparticle surface and also by the cavitation effect of ultrasound irradiation. For the aforementioned mechanism, the significant roles of Co-aminobenzamid@Al-SBA-15, are activation of carbonyl groups and efficient development of the reaction on its high surface area.

**Fig. 6:**
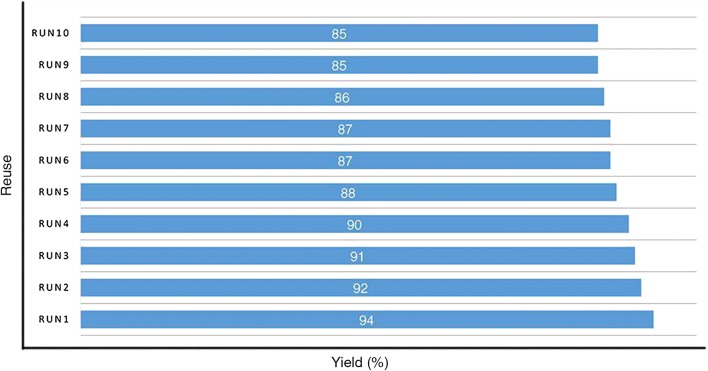
Reusability of Co-aminobenzamid@Al-SBA-15 for the synthesis of 4k

Another distinguishing feature of Co-aminobenzamid@Al-SBA-15 is recoverable without considerable loss of catalytic activity. In order to study the reusability of Co-aminobenzamid@Al-SBA-15 as an environmentally-friendly catalyst, at least ten batches of the experiments were carried out for the preparation of 2,3-diphenyl-2,3-dihydroquinazolin-4(1*H*)-one (4k). To prove this feature, after the accomplishment of the reaction, 5 mL ethanol was added to the reaction mixture and the modified mesoporous was recycled via filtration and washed Co-aminobenzamid@Al-SBA-15 was reused for the new condensation reaction of isatioc anhydride, benzaldehyde and aniline under similar reaction conditions up to ten cycles. There is an insignificant loss of catalytic activity and providing the products in high yield (Fig. 6).

## Conclusions

To recapitulate briefly, we have reported a selective and efficient method for the synthesis of 2,3-dihydroquinazolin-4(1*H*)-ones derivatives via three-component one-pot condensation of isatoic anhydride, aromatic aldehyde and primary amines/or ammonium acetate using Co-aminobenzamid@Al-SBA-15 as a novel catalyst. The current method provides obvious positive points such as environmental friendliness, significantly shorter reaction time, markedly excellent yields and simple workup procedure. In our opinion, we expect this method will find extensive applications in the field of combinatorial chemistry, diversity-oriented synthesis.

## Experimental section

### General

All organic materials were purchased commercially from Sigma-Aldrich, and Merck and were used without further purification. Melting points of products were determined by Electro thermal 9200. All IR spectra were recorded by means of FT-IR Magna spectrometer 550 Nicolet using KBr plates. NMR spectra were attained in DMSO-*d*_*6*_ as a solvent and are reported as parts per million (ppm) downfield from TMS as an internal standard. The NMR spectra were obtained on a Bruker Avance-400 MHz spectrometer. HRMS analyses were carried out using a Bruker micro-TOF-Q-MS analyzer. The elemental analyses (C, H, N) were obtained from a Carlo ERBA Model EA 1108 analyzer. The XRD patterns were recorded on an X-ray diffractometer (PHILIPS, PW 1510, Netherland) using Cu-Kα radiation (λ = 0.154056 nm) in the range 2θ = 0.8–10°. Field Emission Scanning electron microscope (FE-SEM) of nanoparticles was performed on a Model FE-SEM. The particle size and structure were observed using a Philips CM10 transmission electron microscope operating at 30 kV. The N_2_ adsorption/desorption analysis (BET) was performed at 120 °C using an automated gas adsorption analyzer (BEL SORP mini II).

### Preparation of Al-SBA-15

Al-SBA-15 were synthesized following published procedures [31, 32]. In a classic procedure 2 g of Pluronic P123 was dissolved in 75 mL hydrochloric acid solution of pH 1.5. The solution was stirred at 40 °C for 6 h. A second solution was made by adding 3.2 mL of TEOS (tetraethylorthosilicate) and 5 ml of the hydrochloric acid solution of pH 1.5 to a 0.22 g of aluminum triisopropoxide. This suspension was vigorously stirred in a closed flask for 1.5 h during which it became clear. The TEOS solution was quickly added to the surfactant solution and stirred for 20 h at 40 °C. The resulting clear suspension was transferred to a Teflon lined stainless steel autoclave and heated to 100 °C for 20 h. The recovered white solid was filtrated and washed three times with demineralized water and two times with ethanol. The sample was dried overnight at 100 °C. The template was removed by calcination for 10 h at 550 °C with a heating ramp of 1 °C/min. The sample is designated as Al-SBA-15.

### Preparation of APTES@Al-SBA-15

In a 50 mL three-necked round-bottomed flask, 3-aminopropyl triethoxysilane (APTES) (1 mL, 5 mmol) was added dropwise to a suspension of Al-SBA-15 (1 g) in dry toluene (30 mL) under an N_2_ atmosphere. The mixture solution was refluxed for 24 h. After that, the obtained solid was filtered and washed several times with dichloromethane to remove the unreacted starting material, and dried in a vacuum oven at 120 °C for 8 h. The white powder obtained was designated as APTES@Al-SBA-15.

### Preparation of aminobenzamid@Al-SBA-15

**Scheme 3:**
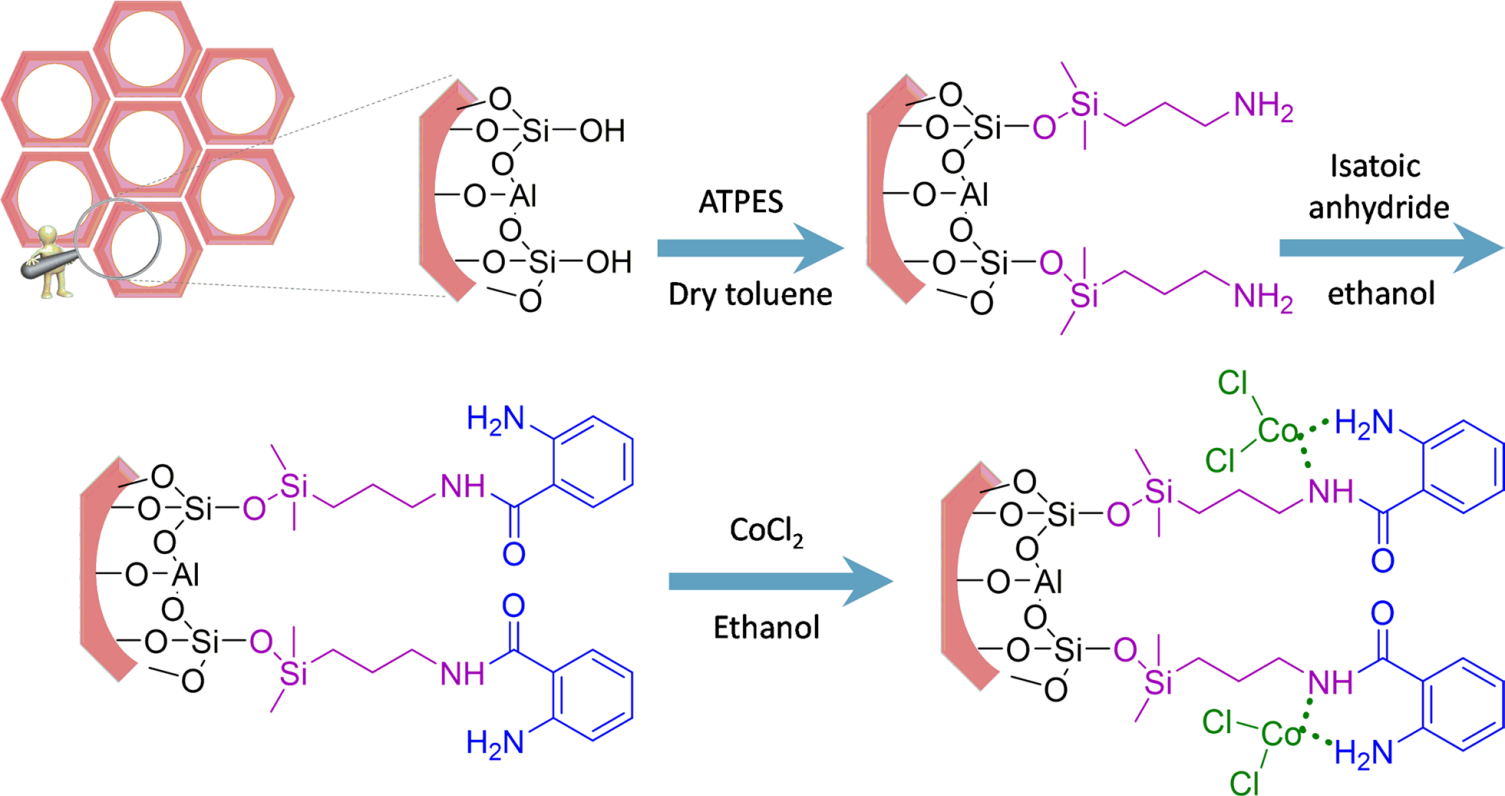
Schematic diagram of preparation Co-aminobenzamid@Al-SBA-15

In a 50 mL round-bottomed flask, isatoic anhydride (0.8 g, 5 mmol) was added to the suspension of APTES@Al-SBA-15 (4 g) in absolute ethanol (30 mL). The mixture solution was refluxed for 12 h. After that, the solid was filtered and washed repeatedly with ice-cold EtOH and dried at room temperature by infrared radiation. The dusty solid obtained was designated as aminobenzamid@Al-SBA-15 (Scheme 3).

### Preparation of Co-aminobenzamid@Al-SBA-15

In a 50 mL round-bottomed flask, aminobenzamid@Al-SBA-15 (0.2 g) was added to the mixture solution of CoCl_2_ (0.1 mmol) in absolute ethanol (25 mL). Afterward, this mixture solution was stirred at room temperature for 12 h, the solid was filtered and washed repeatedly with absolute ethanol until the eluent became colorless, and then dried at room temperature by infrared radiation. The light-green solid obtained was designated as Co-aminobenzamid@Al-SBA-15. The above samples were prepared by the strategy shown in Scheme 3.

### General procedure for the preparation of 2,3-dihydroquinazolin-4(1*H*)-ones under mild reaction

Co-aminobenzamid@Al-SBA-15 as an efficient catalyst was added to an ethanol solution of isatoic anhydride (1 mmol), ammonium acetate (1.2 mmol) or primary aromatic amine (1.1 mmol) and aldehyde (1.0 mmol) were heated in reflux, for desired times. As soon as the complete disappearance of the starting material, as checked by TLC (7:3 n-hexane: ethyl acetate). Then the catalyst was removed by Centrifuging. At that instant, 10 mL ice water was added and the precipitated product was filtered. At the end of the process, the residue was recrystallized from ethanol to obtain the crude product.

### Spectroscopic data for selected compounds

2-(4-Chlorophenyl)-2,3-dihydroquinazolin-4(1*H*)-one **(4c):** m.p. 199–201 °C. FT-IR (KBr, *ν*_max_/cm^−1^): 3307, 3188, 1657, 1609, 1509, 1484. ^1^H NMR (400 MHz, DMSO-*d*_*6*_): δ (ppm) 8.28 (br s, 1H), 7.59 (dd, 1H, *J *= 8.0 Hz, *J *= 1.1 Hz), 7.49 (d, 2H, *J *= 8.6 Hz), 7.44 (d, 2H, *J *= 8.6 Hz), 7.26–7.21–7.26 (m, 1H), 7.12 (br s, 1H), 6.73 (dd, 1H, J = 8.0 Hz, J = 1.1 Hz), 6.67–6.80 (m, 1H), 5.74 (s, 1H). ^13^C NMR (100 MHz, DMSO-d_6_) δ ppm = 161.8, 144.9, 140.1, 131.6, 131.1, 127.7 (2C), 127.5, 126.0, 116.7, 113.2, 113.3, 64.9. HRMS (ESI): m/z [M+H]^+^ calcd for C_14_H_11_N_2_OCl: 258.0559; found: 258.0614. Anal. Calcd. For C_14_H_11_N_2_OCl: C, 65.00; H, 4.29; N, 10.83. Found: C, 65.22; H, 4.19; N, 10.75.

2-(4-Methoxyphenyl)-2,3-dihydroquinazolin-4(1*H*)-one **(4i):** m.p. 177–179 °C. FT-IR (KBr, *ν*_max_/cm^−1^): 3305, 3179, 3054, 1649, 1612, 1508, 1479, 1251, 1037, 749. ^1^H NMR (400 MHz, DMSO-*d*_*6*_): δ (ppm) 8.18 (br s, 1H), 7.62 (d, 1H, *J *= 7.2 Hz), 7.44 (d, 1H, *J *= 7.3 Hz), 7.31 (t, 2H, *J *= 8.6 Hz), 7.20 (t, 1H, *J *= 8.4 Hz), 7.05 (br s, 1H), 6.90 (d, 2H, *J *= 8.6 Hz), 6.71 (t, 1H, *J *= 7.6 Hz), 5.76 (s, 1H), 3.76 (s, 3H). ^13^C NMR (100 MHz, DMSO-*d*_*6*_) δ ppm = 163.5, 158.2, 146.7, 132.9, 132.4, 127.9, 127.3, 116.0, 115.1, 113.7, 113.1, 67.2, 52.9. HRMS (ESI): m/z [M+H]^+^ calcd for C_15_H_14_N_2_O_2_: 254.1055; found 254.1106. Anal. Calcd. For C_15_H_14_N_2_O_2_: C, 70.85; H, 5.55; N, 11.02. Found: C, 70.76; H, 5.59; N, 11.07.

3-(4-Bromophenyl)-2-(4-nitrophenyl)-2,3-dihydroquinazolin-4(1*H*)-one **(4r)** m.p. 237–239 °C. FT-IR (KBr, *ν*_max_/cm^−1^): 3317, 3198, 1662, 1611, 1512, 1474, 762. ^1^H NMR (400 MHz, DMSO-*d*_*6*_): δ (ppm) 8.17 (d, 3H, *J *= 8 Hz), 7.82 (br s, 1H), 7.71(d, 1H, *J *= 8), 7.62 (d, 3H, *J *= 8 Hz), 7.53 (d, 2H, *J *= 8 Hz), 7.25 (t, 2H, *J *= 12), 6.77–6.72 (m, 1H), 6.48 (s, 1H). ^13^C NMR (100 MHz, DMSO-*d*_*6*_) δ ppm = 165.6, 161.2, 146.3, 134.4, 132.2, 129.9, 128.2, 125.6, 122.8, 118.1, 117.2, 116.9, 115.3, 113.6, 68.9, 59.3. HRMS (ESI): m/z [M+H]^+^ calcd for C_20_H_14_N_3_O_3_Br: 423.0218; found: 423.0262. Anal. calcd. for C_20_H_14_N_3_O_3_Br: C, 56.73; H, 3.30; N, 9.92, Found: C, 56.58; H, 3.36; N, 9.82.

3-Benzyl-2-(4-nitrophenyl)-2,3-dihydroquinazolin-4(1*H*)-one **(4s)** m.p. 191–192 °C. FT-IR (KBr, *ν*_max_/cm^−1^): 3412, 3017, 2332, 1592, 1512, 1425, 774. ^1^H NMR (400 MHz, DMSO-*d*_*6*_): δ (ppm) 8.18(d, *J *= 8, 1H), 7.69(d, *J *= 8, 1H), 7.55 (d, 2H, *J *= 8), 7.31–7.20 (m, 8H), 6.72–6.63 (m, 2H), 5.95 (d, 1H, *J *= 4 Hz), 5.29 (d, 1H, *J *= 12 Hz), 3.96 (d, *J *= 12 Hz, 1H). ^13^C NMR (100 MHz, DMSO-*d*_*6*_) δ ppm = 163.1, 148.4, 141.1, 133.0, 132.4, 128.5, 125.2(2C), 123.8, 123.2, 121.3, 117.5, 113.9, 114.8, 114.2, 68.1, 52.7. HRMS (ESI): m/z [M+H]^+^ calcd for C_21_H_17_N_3_O_3_: 359.1269; found: 359.1311. Anal. calcd. for C_21_H_17_N_3_O_3_: C, 68.29; H, 4.60; N, 11.38, Found: C, 68.19; H, 4.51; N, 11.22.

## Additional file

**Additional file 1.** Additional information.
