# Supplementary material for: Co-aminobenzamid@Al-SBA-15: a favorable catalyst in synthesis of 2,3-dihydroquinazolin-4(1H)-ones
Source: BMC Chem. 2019 Feb 28;13(1):26. doi: 10.1186/s13065-019-0517-7 (PMC6659566; doi:10.1186/s13065-019-0517-7)
Supplement: Supplementary file 1 — Additional file 1. Additional information. [file 13065_2019_517_MOESM1_ESM.docx]

**Co-aminobenzamid@Al-SBA-15: A favorable catalyst in synthesis of 2,3-dihydroquinazolin-4(1*H*)-ones**

Javad Safaei-Ghomi^*^, Raheleh Teymuri

*Department of Organic Chemistry, Faculty of Chemistry, University of Kashan, Kashan, P.O. Box 87317-51167, I. R. Iran Corresponding author. E-mail addresses:* [*safaei@kashanu.ac.ir*](mailto:safaei@kashanu.ac.ir)*, Fax:* ***+****98-31-55912397; Tel.: +98-31-55912385*

**General procedure for the preparation of 2,3-dihydroquinazolin-4(1*H*)-ones under mild reaction:**

Co‐aminobenzamid@Al-SBA-15 as an efficient catalyst was added to an ethanol solution of isatoic anhydride (1 mmol), ammonium acetate (1.2 mmol) or primary aromatic amine (1.1 mmol) and aldehyde (1.0 mmol) were heated in reflux, for desired times. As soon as the complete disappearance of the starting material, as checked by TLC (7:3 n-hexane: ethyl acetate). Then the catalyst was removed by Centrifuging. At that instant, 10 ml ice water was added and the precipitated product was filtered. At the end of the process, the residue was recrystallized from ethanol to obtain the crude product.

**Characterization of compounds:**

**2-Phenyl-2,3-dihydroquinazolin-4(1*H*)-one (4a):** m.p. 219-221 °C, FT-IR (KBr, *ν*_max_ /cm^-1^): 3309, 3187, 1661, 1602, 1510, 1482. ^1^H NMR (400 MHz, DMSO-*d_6_*): δ (ppm) 8.26 (br s, 1H), 7.59 (d, 1H, *J*= 7.8 Hz), 7.47 (d, 2H, *J*= 7.1 Hz), 7.39-7.33 (m, 3H), 7.22 (t, 1H, *J*= 7.8 Hz), 7.09 (br s, 1H), 6.73 (d, 1H, *J*= 7.8 Hz), 6.65 (t, 1H, *J*= 7.8 Hz), 5.73 (s, 1H). ^13^C NMR (100 MHz, DMSO-d_6_) δ ppm = 162.6, 145.7, 140.4, 132.1, 127.3, 127.9 (2C), 126.3, 124.7 (2C), 116.5, 113.6, 112.8, 65.2. HRMS (ESI): m/z [M +H]^+^ calcd for C_14_H_12_N_2_O: 224.0949; found 224.0921. Anal. Calcd. For C_14_H_12_N_2_O: C, 74.98; H, 5.39; N, 12.49. Found: C, 74.67; H, 5.43; N, 12.38.

**2-(4-Nitrophenyl)-2,3-dihydroquinazolin-4(1*H*)-one (4b):** m.p. 214-215 °C, FT-IR (KBr, *ν*_max_ /cm^-1^): 3431, 3281, 1646, 1610, 1520, 1485, 1347. ^1^H NMR (400 MHz, DMSO-*d_6_*): δ (ppm) 8.51 (br s, 1H), 8.27 (d, 2H, *J*= 8.7 Hz), 7.72 (d, 2H, *J*= 8.7 Hz), 7.59 (dd, 1H, *J*= 7.9 Hz, *J*= 1.6 Hz), 7.32 (br s, 1H), 7.25 (t, 1H), 6.75 (d, 1H, *J*= 7.9 Hz), 6.68 (t, 1H), 5.89 (s, 1H). ^13^C NMR (100 MHz, DMSO-d_6_) δ ppm = 161.8, 148.2, 146.4, 147.1, 131.6, 127.1, 126.2, 122.9, 116.8, 112.9, 112.1, 63.9. HRMS (ESI): m/z [M +H]^+^ calcd for C_14_H_11_N_3_O_3_: 269.0800; found 269.0862. Anal. Calcd. For C_14_H_11_N_3_O_3_: C, 62.48; H, 4.08; N, 15.60. Found: C, 62.26; H, 4.17; N, 15.51.

**2-(4-Chlorophenyl)-2,3-dihydroquinazolin-4(1*H*)-one (4c):** m.p. 199-201 °C. FT-IR (KBr, *ν*_max_ /cm^-1^): 3307, 3188, 1657, 1609, 1509, 1484. ^1^H NMR (400 MHz, DMSO-*d_6_*): δ (ppm) 8.28 (br s, 1H), 7.59 (dd, 1H, *J*= 8.0 Hz, *J*= 1.1 Hz), 7.49 (d, 2H, *J*= 8.6 Hz), 7.44 (d, 2H, *J*= 8.6 Hz), 7.26-7.21-7.26 (m, 1H), 7.12 (br s, 1H), 6.73 (dd, 1H, J= 8.0 Hz, J= 1.1 Hz), 6.67-6.80 (m, 1H), 5.74 (s, 1H). ^13^C NMR (100 MHz, DMSO-d_6_) δ ppm = 161.8, 144.9, 140.1, 131.6, 131.1, 127.7 (2C), 127.5, 126.0, 116.7, 113.2, 113.3, 64.9. HRMS (ESI): m/z [M +H]^+^ calcd for C_14_H_11_N_2_OCl: 258.0559; found: 258.0614. Anal. Calcd. For C_14_H_11_N_2_OCl: C, 65.00; H, 4.29; N, 10.83. Found: C, 65.22; H, 4.19; N, 10.75.

**2-(p-Tolyl)-2,3-dihydroquinazolin-4(1*H*)-one (4d):** m.p. 233-235 °C. FT-IR (KBr, *ν*_max_ /cm^-1^): 3314, 3186, 1662, 1603, 1507, 1469. ^1^H NMR (400 MHz, DMSO-*d_6_*): δ (ppm) 8.31 (br s, 1H), 7.61 (d, 1H, *J*=7.8 Hz), 7.29 (d, 2H, *J*=8.0 Hz), 7.19–7.26 (m, 3H), 7.10 (s, 1H), 6.59 (d, 1H, *J*= 8.0 Hz), 6.50 (t, 1H, J =7.8 Hz), 5.39 (s, 1H), 2.24 (s, 3H). ^13^C NMR (100 MHz, DMSO-d_6_) δ ppm = 164.6, 147.1, 137.5, 136.2, 132.4, 128.5, 126.7, 126.4, 115.1, 114.8, 113.9, 67.2, 21.3. HRMS (ESI): m/z [M +H]^+^ calcd for C_15_H_14_N_2_O: 238.1106; found 238.1158. Anal. Calcd. For C_15_H_14_N_2_O: C, 75.63; H, 5.88; N, 11.76. Found: C, 75.68; H, 5.75; N, 11.58.

**2-(2-Nitrophenyl)-2,3-dihydroquinazolin-4(1*H*)-one (4e):** m.p. 192-193 °C. FT-IR (KBr, *ν*_max_ /cm^-1^): 3309, 3189, 1647, 1611, 1521, 1472. ^1^H NMR (400 MHz, DMSO-*d_6_*): δ (ppm) 8.82 (br s, 1H), 8.15 (d, 1H, *J*= 8.6 Hz), 7.91-7.79 (m, 4H), 7.56 (t, 2H, *J*= 8 Hz), 7.36 (t, 1H, *J*=7.2), 7.18 (d, 1H, *J*=7.6 Hz), 6.31 (br s, 1H). ^13^C NMR (100 MHz, DMSO-*d_6_*) δ ppm = 164.1, 148.2, 146.8, 145.3, 133.5, 133.1, 132.8, 128.1, 126.8, 122.6, 118.2, 114.8, 114.5, 65.2. HRMS (ESI): m/z [M +H]^+^ calcd for C_14_H_11_N_3_O_3_: 258.0800; found 258.0904. Anal. Calcd. For C_14_H_11_N_3_O_3_: C, 62.45; H, 4.09; N, 15.61. Found: C, 62.39; H, 4.02; N, 15.72.

**2-(2-Chlorophenyl)-2,3-dihydroquinazolin-4(1*H*)-one (4f):** m.p. 202-204 °C. FT-IR (KBr, *ν*_max_ /cm^-1^): 3364, 3181, 1652, 1503, 1398, 751. ^1^H NMR (400 MHz, DMSO-*d_6_*): δ (ppm) 8.19 (br s, 1H), 7.62 (d, *J*= 6.0 Hz, 2H), 7.50–7.46 (m, 1H), 7.42–7.38 (m, 2H), 7.25 (t, 1H, *J*=8.4 Hz), 7.10 (br s, 1H), 6.53 (d, 1H, *J*=8.0 Hz), 6.69 (t, 1H, *J*=7.2 Hz), 6.13 (s, 1H), ^13^C NMR (100 MHz, DMSO-*d_6_*) δ ppm = 162.1, 145.9, 138.9, 134.1, 130.2, 129.8, 129.1, 127.3, 126.6, 125.7, 118.3, 115.3, 114.1, 64.6. HRMS (ESI): m/z [M +H]^+^ calcd for C_14_H_11_N_2_OCl: 258.0559; found: 259.0643. Anal. Calcd. For C_14_H_11_N_2_OCl: C, 65.00; H, 4.29; N, 10.83. Found: C, 65.21; H, 4.34; N, 10.72.

**2-(3-Methylphenyl)-2,3-dihydroquinazolin-4(1*H*)-one (4g):** m.p. 225-226 °C. FT-IR (KBr, *ν*_max_ /cm^-1^): 3307, 3188, 1657, 1609, 1509, 1484. ^1^H NMR (400 MHz, DMSO-*d_6_*): δ (ppm) 8.29 (br s, 1H), 7.59 (d, 1H, *J*= 7.6 Hz), 7.28-7.31 (m, 1H), 7.25-7.21 (m, 1H), 7.12 (br s, 1H), 7.03 (s, 2H), 6.91-6.87 (m, 1H), 6.75-6.73 (m, 1H), 6.66-6.64 (m, 1H), 5.70 (s, 1H), 3.32 (s, 3H). ^13^C NMR (100 MHz, DMSO-*d_6_*) δ ppm = 163.1, 147.7, 137.4, 135.2, 132.6, 131.8, 127.9, 126.8, 124.3, 117.5, 115.6, 114.2, 62.8, 47.3, 19.2. HRMS (ESI): m/z [M +H]^+^ calcd for C_15_H_14_N_2_O: 238.1106; found 238.1173. Anal. Calcd. For C_15_H_14_N_2_O: C, 75.61; H, 5.92; N, 11.76. Found: C, 75.54; H, 6.02; N, 11.69.

**2-(4-bromophenyl)-2,3-dihydroquinazolin-4(1*H*)-one (4h):** m.p. 203-204 °C. FT-IR (KBr, *ν*_max_ /cm^-1^): 3407, 3158, 1638, 1601, 1511, 1466. ^1^H NMR (400 MHz, DMSO-*d_6_*): δ (ppm) 8.15 (br s, 1H), 7.62 (dd, 1H, *J*= 8.0 Hz, *J*= 1.1 Hz), 7.51 (d, 2H, *J*= 8.6 Hz), 7.33 (d, 2H, *J*= 8.6 Hz), 7.27-7.18-7.23 (m, 1H), 7.11 (br s, 1H), 6.61 (dd, 1H, *J*= 8.0 Hz, *J*= 1.1 Hz), 6.59-6.43 (m, 1H), 5.12 (s, 1H). ^13^C NMR (100 MHz, DMSO-*d_6_*) δ ppm = 162.6, 143.7, 141.9, 132.3, 130.8, 127.4, 125.2, 124.7, 122.0, 115.4, 112.9, 110.2, 65.1. HRMS (ESI): m/z [M +H]^+^ calcd for C_14_H_11_N_2_OBr: 302.0054; found 302.0123. Anal. Calcd. For C_14_H_11_N_2_OBr: C, 55.47; H, 3.66; N, 9.24. Found: C, 55.32; H, 3.73; N, 9.21.

**2-(4-Methoxyphenyl)-2,3-dihydroquinazolin-4(1*H*)-one (4i):** m.p. 177-179 °C. FT-IR (KBr, *ν*_max_ /cm^-1^): 3305, 3179, 3054, 1649, 1612, 1508, 1479, 1251, 1037, 749. ^1^H NMR (400 MHz, DMSO-*d_6_*): δ (ppm) 8.18 (br s, 1H), 7.62 (d, 1H, *J*= 7.2 Hz), 7.44 (d, 1H, *J*= 7.3 Hz), 7.31 (t, 2H, *J*=8.6 Hz), 7.20 (t, 1H, *J*=8.4 Hz), 7.05 (br s, 1H), 6.90 (d, 2H, *J*=8.6 Hz), 6.71 (t, 1H, *J*=7.6 Hz), 5.76 (s, 1H), 3.76 (s, 3H). ^13^C NMR (100 MHz, DMSO-*d_6_*) δ ppm = 163.5, 158.2, 146.7, 132.9, 132.4, 127.9, 127.3, 116.0, 115.1, 113.7, 113.1, 67.2, 52.9. HRMS (ESI): m/z [M +H]^+^ calcd for C_15_H_14_N_2_O_2_: 254.1055; found 254.1106. Anal. Calcd. For C_15_H_14_N_2_O_2_: C, 70.85; H, 5.55; N, 11.02. Found: C, 70.76; H, 5.59; N, 11.07.

**2-(4-(dimethylamino) phenyl)-2,3-dihydroquinazolin-4(1*H*)-one (4j):** m.p. 209-211 °C. FT-IR (KBr, *ν*_max_ /cm^-1^): 3305, 3179, 3054, 1649, 1612, 1508, 1479, 1251, 1037, 749. ^1^H NMR (400 MHz, DMSO-*d_6_*): δ (ppm) 8.68 (br s, 1H), 7.91 (d, 1H, *J*= 8.2 Hz), 7.58 (t, 1H,  *J*=8.1 Hz), 7.41 (d, 2H, *J*= 7.9 Hz), 7.17 (d, 1H, *J*= 7.2 Hz),7.9-7.16 (m, 1H), 6.72 (d, 2H, *J*=7.9 Hz), 6.41 (br s, 1H), 5.82 (s, 1H) 3.13 (s, 6H). ^13^C NMR (100 MHz, DMSO-*d_6_*) δ ppm= 158.1, 153.4, 149.8, 127.6, 127.2, 125.4, 124.8, 123.7, 120.0, 119.1, 111.9, 75.3, 40.1. HRMS (ESI): m/z [M +H]^+^ calcd for C_16_H_17_N_3_O: 267.1371; found 267.1310. Anal. Calcd. For C_16_H_17_N_3_O: C, 71.92; H, 6.36; N, 15.72. Found: C, 72.05; H, 6.39; N, 15.64.

**2,3-diphenyl-2,3-dihydroquinazolin-4(1*H*)-one (4k):** m.p. 205-207 °C. FT-IR (KBr, *ν*_max_ /cm^-1^): 3294, 3059, 1633, 1510, 1391, 1158, 7752, 696. ^1^H NMR (400 MHz, DMSO-*d_6_*): δ (ppm) 7.71 (d, 1H, *J*= 7.9 Hz), 7.35–7.38 (m, 2H), 7.24–7.31 (m, 6H), 7.14–7.23 (m, 3H), 6.88–6.91 (m, 1H), 6.77 (d, 1H, *J*= 8.0 Hz), 6.71 (s, 1H), 6.25 (s, 1H). ^13^C NMR (100 MHz, DMSO-*d_6_*) δ ppm = 161.9, 144.8, 140.78, 140.4, 132.1, 127.1, 126.6, 127.2, 126.8, 124.3, 125.2, 124.9, 116.3, 114.1, 112.8, 70.7. HRMS (ESI): m/z [M +H]^+^ calcd for C_20_H_16_N_2_O: 300.1262; found: 300.1209. Anal. Calcd. For C_20_H_16_N_2_O: C, 80.01; H, 5.32; N, 9.32. Found: C, 79.82; H, 5.41; N, 9.18,

**2-(4-nitrophenyl)-3-phenyl-2,3-dihydroquinazolin-4(1*H*)-one (4l):** m.p. 193-195 °C. FT-IR (KBr, *ν*_max_ /cm^-1^): 3274, 1633, 1521, 1347, 752. ^1^H NMR (400 MHz, DMSO-*d_6_*): δ (ppm) 8.02 (d, 2H, *J*=8.7 Hz), 7.89 (dd, 1H, *J*=7.9, 1.4 Hz), 7.46(d, 2H, *J*=8.7 Hz), 7.21 (t, 3H, *J*=7.5 Hz), 7.16 (dd, 3H, *J*= 7.5, 6.2 Hz), 6.87-6.79 (m, 1H), 6.68 (d, 1H, *J*= 8.0 Hz), 6.18 (s, 1H), 4.56 (s, 1H) ^13^C NMR (100 MHz, DMSO-*d_6_*) δ ppm = 157.6, 132.8, 131.5, 129.1, 128.9, 128.3, 127.1, 126.2, 125.8, 125.1, 122.3(2C), 120.9, 116.5, 114.4, 68.7. HRMS (ESI): m/z [M +H]^+^ calcd for C_20_H_15_N_3_O_3_: 345.1113; found 345.1209. Anal. Calcd. For C_20_H_15_N_3_O_3_: C, 69.58; H, 4.34; N, 12.16. Found: C, 69.71; H, 4.11; N, 11.93.

**2-(4-chlorophenyl)-3-phenyl-2,3-dihydroquinazolin-4(1*H*)-one (4m):** m.p. 217-219 °C. FT-IR (KBr, *ν*_max_ /cm^-1^): 3308, 1651, 1642, 1612, 1504, 1432. ^1^H NMR (400 MHz, DMSO-*d_6_*): δ (ppm) 8.20 (d, 1H, *J*= 7.9 Hz), 7.34–7.42 (m, 2H), 7.22–7.28 (m, 5H), 7.14–7.19 (m, 3H), 6.74–6.78 (m, 1H), 6.56 (d, 1H, *J*= 8.1 Hz), 6.01 (br s, 1H), 4.58 (s, 1H). ^13^C NMR (100 MHz, DMSO-*d_6_*) δ ppm = 161.3, 144.6, 132.7, 130.4, 126.6, 125.7, 124.5(2C), 123.9, 120.8, 120.1(2C), 119.8, 113.1, 112.2, 65.3. HRMS (ESI): m/z [M +H]^+^ calcd for C_20_H_15_N_2_OCl: 334.0872; found: 334.0861. Anal. Calcd. For C_20_H_15_N_2_OCl: C, 71.74; H, 4.52; N, 8.37. Found: C, 71.65; H, 4.71; N, 8.24.

**3-phenyl-2-(p-tolyl)-2,3-dihydroquinazolin-4(1*H*)-one (4n):** m.p. 212-214 °C. FT-IR (KBr, *ν*_max_ /cm^-1^): 3301, 3179, 1654, 1603, 1510, 1465. ^1^H NMR (400 MHz, DMSO-*d_6_*): δ (ppm) 8.18 (d, 1H, J= 7.6 Hz), 7.54–7.50 (m, 2H), 7.48–7.35 (m, 6H), 7.09-7.06 (m, 1H), 6.83 (d, 1H, *J*= 8 Hz), 6.63 (d, 2H, *J*= 8.2 Hz), 6.05 (s, 1H), 4.69 (s, 1H), 2.12 (s, 3H) ^13^C NMR (100 MHz, DMSO-*d_6_*) δ ppm = 164.8, 149.2, 134.0, 127.3 126.7, 125.6(2C), 124.5, 123.9, 122.7, 120.8, 117.4, 116.2, 113.6, 109.3, 72.9, 19.8. HRMS (ESI): m/z [M +H]^+^ calcd for C_20_H_18_N_2_O: 315.14919; found: 315.1484. Anal. Calcd. For C_20_H_18_N_2_O: C, 79.48; H, 5.95; N, 9.26. Found: C, 79.45; H, 6.08; N, 9.18.

**2-(3-nitrophenyl)-3-phenyl-2,3-dihydroquinazolin-4(1*H*)-one (4o):** m.p. 184-185 °C. FT-IR (KBr, *ν*_max_ /cm^-1^): 3272, 1628, 1525, 1332, 751. ^1^H NMR (400 MHz, DMSO-*d_6_*): δ (ppm) 8.45 (d, 1H, *J*= 7.9 Hz), 7.74–7.70 (m, 2H), 7.55–7.49 (m, 2H), 7.37–7.30 (m, 6H), 6.61–6.52 (m, 1H), 6.48 (d, 1H, *J*= 7.9 Hz), 6.10 (s, 1H), 4.42 (s, 1H). ^13^C NMR (100 MHz, DMSO-*d_6_*) δ ppm = 160.4, 147.2, 131.9, 126.3(2C), 123.8(2C), 121.8, 120.5, 115.2, 113.6, 112.5, 70.3, 33.4, 17.4, 14.2. HRMS (ESI): m/z [M +H]^+^ calcd for C_20_H_15_N_3_O_3_: 345.1113; found: 345.2192. Anal. Calcd. For C_20_H_15_N_3_O_3_: C, 69.58; H, 4.34; N, 12.16. Found: C, 69.73; H, 4.12; N, 11.98.

**2-(4-bromophenyl)-3-phenyl-2,3-dihydroquinazolin-4(1*H*)-one (4p):** m.p. 216-218 °C. FT-IR (KBr, *ν*_max_ /cm^-1^): 3349, 1663, 1606, 1517, 1456. ^1^H NMR (400 MHz, DMSO-*d_6_*): δ (ppm) 7.73-7.62 (m, 2H), 7.55-7.48 (m, 2H), 7.33-7.11 (m, 8H), 6.81-6.76 (m, 2H), 6.26 (d, 1H, *J*= 3.8 Hz); ^13^C NMR (100 MHz, DMSO-*d_6_*) δ ppm = 161.8, 145.3, 140.7, 139.9, 133.3, 131.2, 128.4, 127.9, 127.2, 125.4, 122.3, 120.6, 117.5, 115.2, 114.7, 73.3. HRMS (ESI): m/z [M +H]^+^ calcd for C_20_H_15_N_2_OBr: 378.0367; found: 378.0314. Anal. Calcd. For C_20_H_15_N_2_OBr: C, 63.34; H, 3.99; N, 7.39. Found: C, 62.53; H, 4.19; N, 7.51.

**2-(4-nitrophenyl)-3-(p-tolyl)-2,3-dihydroquinazolin-4(1*H*)-one (4q):** m.p. 211-213 °C. FT-IR (KBr, *ν*_max_ /cm^-1^): 3,650, 3,030, 2,361, 1,660, 1,594, 1,515, 1,457, 762. ^1^H NMR (400 MHz, DMSO-*d_6_*): δ (ppm) 10.22 (s, 1H), 8.76 (s, 1H), 8.34 (d, 2H, *J*= 8.6 Hz), 8.17 (d, 2H, *J*= 8.6 Hz), 7.72 (dd, 1H, *J*= 1.0, 6.4 Hz,), 7.60–7.54 (m, 3H), 7.38 (t, 1H, *J*= 7.6 Hz,), 7.29 (d, 1H,  *J*= 7.8 Hz,), 7.11 (d, 2H, J= 8.2 Hz,), 2.48 (s, 3H). ^13^C NMR (100 MHz, DMSO-*d_6_*) δ ppm = 161.3, 148.8, 147.6, 140.5, 135.4, 133.4, 132.1, 130.4, 128.9, 129.1, 128.6, 125.8, 123.4, 118.4, 118.0, 73.1, 21.2. HRMS (ESI): m/z [M +H]^+^ calcd for C_21_H_17_N_3_O_3_: 359.1269; found: 359.1311. Anal. Calcd. For C_21_H_17_N_3_O_3_: C, 70.21; H, 4.73; N, 11.69. Found: C, 70.45; H, 4.98; N, 11.51.

**3-(4-bromophenyl)-2-(4-nitrophenyl)-2,3-dihydroquinazolin-4(1*H*)-one (4r)** m.p. 237-239 °C. FT-IR (KBr, *ν*_max_ /cm^-1^): 3317, 3198, 1662, 1611, 1512, 1474, 762. ^1^H NMR (400 MHz, DMSO-*d_6_*): δ (ppm) 8.17 (d, 3H, *J*=8 Hz), 7.82 (br s, 1H), 7.71(d, 1H, *J*=8), 7.62 (d, 3H, *J*= 8 Hz), 7.53 (d, 2H, *J*=8 Hz), 7.25 (t, 2H, *J*=12), 6.77-6.72 (m, 1H), 6.48 (s, 1H). ^13^C NMR (100 MHz, DMSO-*d_6_*) δ ppm = 165.6, 161.2, 146.3, 134.4, 132.2, 129.9, 128.2, 125.6, 122.8, 118.1, 117.2, 116.9, 115.3, 113.6, 68.9, 59.3. HRMS (ESI): m/z [M +H]^+^ calcd for C_20_H_14_N_3_O_3_Br: 423.0218; found: 423.0262. Anal. calcd. for C_20_H_14_N_3_O_3_Br: C, 56.73; H, 3.30; N, 9.92, Found: C, 56.58; H, 3.36; N, 9.82.

**3-benzyl-2-(4-nitrophenyl)-2,3-dihydroquinazolin-4(1*H*)-one (4s)** m.p. 191-192 °C. FT-IR (KBr, *ν*_max_ /cm^-1^): 3412, 3017, 2332, 1592, 1512, 1425, 774. ^1^H NMR (400 MHz, DMSO-*d_6_*): δ (ppm) 8.18(d, *J*=8, 1H), 7.69(d, *J*=8, 1H), 7.55 (d, 2H, *J*= 8), 7.31–7.20 (m, 8H), 6.72–6.63 (m, 2H), 5.95 (d, 1H, *J*= 4 Hz), 5.29 (d, 1H, *J*= 12 Hz), 3.96 (d, *J*= 12 Hz, 1H). ^13^C NMR (100 MHz, DMSO-*d_6_*) δ ppm = 163.1, 148.4, 141.1, 133.0, 132.4, 128.5, 125.2(2C), 123.8, 123.2, 121.3, 117.5, 113.9, 114.8, 114.2, 68.1, 52.7. HRMS (ESI): m/z [M +H]^+^ calcd for C_21_H_17_N_3_O_3_: 359.1269; found: 359.1311. Anal. calcd. for C_21_H_17_N_3_O_3_: C, 68.29; H, 4.60; N, 11.38, Found: C, 68.19; H, 4.51; N, 11.22.

**3-benzyl-2-phenyl-2,3-dihydroquinazolin-4(1*H*)-one (4t):** m.p. 156-157 °C. FT-IR (KBr, *ν*_max_ /cm^-1^): 3411, 3019, 2354, 1634, 1581, 1511, 1432, 757. ^1^H NMR (400 MHz, DMSO-*d_6_*): δ (ppm) 7.91 (dd, 1H, *J*= 1.4, 6.4 Hz), 7.33–7.19 (m, 12H), 6.54–6.48 (m, 2H), 5.49 (d, 1H, *J*= 2.6 Hz), 5.55 (d, 1H, *J*= 15.4 Hz), 3.31 (d, 1H *J*= 15.4 Hz). ^13^C NMR (100 MHz, DMSO-*d_6_*) δ ppm = 164.1, 142.8, 140.1, 135.9, 133.7, 129.6, 127.9, 126.8, 125.1(2C), 124.3, 122.7, 118.1, 115.5, 113.2, 70.1, 49.4. HRMS (ESI): m/z [M +H]^+^ calcd for C_21_H_18_N_2_O: 314.1419; found: 314.1436. Anal. Calcd. For C_21_H_18_N_2_O: C, 80.23; H, 5.77; N, 8.91. Found: C, 79.45; H, 5.98; N, 8.75.
